# Implementation of ERAS guidelines in patients undergoing CRS and HIPEC: need for multicentre trial

**DOI:** 10.1515/pp-2023-0022

**Published:** 2023-12-20

**Authors:** Christos Iavazzo, Ioannis D. Gkegkes, John Spiliotis

**Affiliations:** Gynaecological Oncology Department, Metaxa Cancer Hospital, Piraeus, Greece; Athens Colorectal Laboratory, Athens, Greece; Department of Colorectal Surgery, Royal Devon and Exeter NHS Foundation Trust, Exeter, UK; Department of Surgical Oncology and HIPEC, European Interbalkan Medical Center, Thessaloniki, Greece

**Keywords:** HIPEC, enhanced recovery after surgery (ERAS) protocoll, cytoreductive surgery (CRS)

To the Editor,

With great deal of interest we read the article entitled: “Implementation of an enhanced recovery program for complete cytoreductive surgery and hyperthermic intraperitoneal chemotherapy in a referral center: a case control prospective study” by Charleux-Muller et al. [1].

The authors present their initial experience with enhanced recovery after surgery (ERAS) implementation in a small cohort of patients undergoing cytoreductive surgery (CRS)/Hyperthermic IntraPEritoneal Chemotherapy (HIPEC) and show that ERAS can decrease both ICU stay, and hospital stay as well as morbidity. Interestingly, patients’ compliance was >95 %. Recently, ERAS Society published guidelines for patients undergoing CRS and HIPEC [2, 3]. We would like to thank the authors for sharing their valuable experience and protocol, especially in the postoperative period.

We would like to highlight the findings of a recent metanalysis in the field, including seven studies, which revealed decrease in hospital stay, readmission as well as high levels of safety and compliance, even though ERAS was analysed in heterogeneous patients and surgical pathology as well as in different settings [4]. One of the limitations of this systematic review was that no RCTs were included in the analysis.

We would like to propose a multicentre cooperation among centres of excellence in CRS and HIPEC in order to implement the newly published ERAS guidelines in a larger number of patients and in different settings. Such implementation can improve patients’ postoperative recovery and furthermore can decrease the cost of such interventions both for the patients as well as the national health systems. We would like to highlight that there is already an ongoing observational trial (NCT05185791) on the same subject [5], a fact that could potentially lead to research duplication. Instead, it might be more prudent to suggest that another research team may undertake such a trial, providing an opportunity for external validation of the results in order to enhance the value of such a prospective study, even though it may pose some challenges in terms of execution.

Once again, we would like to thank the authors for sharing their valuable experience.
